# Pediatric Heart Failure: A Practical Guide for Primary Care Providers Supporting Families Across the Care Continuum

**DOI:** 10.3390/children12101293

**Published:** 2025-09-24

**Authors:** Shahnawaz Amdani, Kriti Puri, Julie Glickstein, Joseph A. Spinner, Jonathan N. Johnson, Ashraf S. Harahsheh, Majd Makhoul, Susan Denfield

**Affiliations:** 1 Division of Cardiology & Cardiovascular Medicine, Children’s Institute, Department of Heart Vascular & Thoracic, Cleveland, OH 44195, USA; 2 Divisions of Critical Care and Cardiology, Department of Pediatrics, Baylor College of Medicine, Houston, TX 77030, USA; kriti.puri@bcm.edu; 3 Morgan Stanley Children’s Hospital New York-Presbyterian, Columbia University Irving Medical Center, New York, NY 10032, USA; jg2065@cumc.columbia.edu; 4 Division of Cardiology, Department of Pediatrics, Baylor College of Medicine, Texas Children’s Hospital, Houston, TX 77030, USA; spinner@bcm.edu; 5 Division of Pediatric Cardiology, Department of Pediatric and Adolescent Medicine, Mayo Clinic, Rochester, MN 55905, USA; johnson.jonathan@mayo.edu; 6 Division of Cardiology, Department of Pediatrics, Children’s National Hospital, The George Washington University School of Medicine & Health Sciences, Washington, DC 20010, USA; aharahsh@childrensnational.org; 7 Division of Pediatric Cardiology, Atrium Health Levine Children’s Hospital, Charlotte, NC 77030, USA; majd.makhoul@atriumhealth.org; 8 Baylor College of Medicine, Houston, TX 77030, USA; denfield@bcm.edu

**Keywords:** pediatric heart failure, cardiomyopathy, congenital heart disease, family-centered care, ventricular assist device, heart transplant, primary care pediatrics

## Abstract

**Background/Objectives**: Pediatric heart failure (HF) is a complex clinical syndrome with diverse etiologies and presentations. Primary care providers (PCPs) are critical in early recognition, family education, and coordination of ongoing care in collaboration with pediatric cardiologists. This review provides a comprehensive guide for PCPs to understand the causes, symptoms, treatments, and longitudinal considerations for children with HF. **Methods**: We conducted a narrative review synthesizing published evidence and expert consensus guidelines, focusing on the clinical presentation, treatment options, and longitudinal care strategies in children with HF. **Results**: HF in children most commonly results from congenital heart disease and cardiomyopathy, but includes a wide range of etiologies. Presenting symptoms differ by age, with subtle signs often mistaken for common pediatric illnesses. Treatments range from disease-modifying medications to surgical and transplant therapies. Psychosocial support, mental health, and family-centered care are critical components of long-term management. **Conclusions**: PCPs are essential partners in the care of children with HF. A proactive, collaborative, and informed approach can improve quality of life, reduce hospitalizations, and support families navigating this challenging diagnosis.

## 1. Introduction

Hospitalizations for children with heart failure (HF) have been increasing in frequency since the early 2000s [1,2]. The etiology of HF in children is diverse and age-dependent, leading to varied prognoses and often confusion among families. It is not uncommon for parents to compare treatment approaches with those of other families and struggle to understand why management differs for their child. This review aims to equip pediatric practitioners with a comprehensive framework to support families in understanding the signs, etiologies, and management strategies associated with pediatric HF. Collaborative care involving both general pediatricians and pediatric cardiology subspecialists is essential to navigating the complex medical and psychosocial aspects of this condition.

## 2. Causes of Pediatric Heart Failure

Pediatric HF encompasses a wide range of etiologies, with the most common causes being congenital heart disease (CHD) and intrinsic myocardial dysfunction [2,3,4]. Intrinsic myocardial dysfunction may be due to cardiomyopathies, arrhythmia-induced, infections, and inflammatory or metabolic diseases (Table 1), and the most likely causes vary with age (Table 2). Identifying the underlying etiology is key to planning treatment strategies and requires a detailed clinical evaluation, including history and physical examination, starting with the pediatric practitioner and subsequent referral and targeted investigations.

In infants, unrepaired CHDs are frequent causes of HF [2,5]. High-output states like vein of Galen malformations also represent rare but important etiologies.

In older children and adolescents, CHD remains a common etiology, often in the context of previously diagnosed or palliated lesions. Patients with a single ventricle, as well as those with two anatomically well-formed ventricles, may develop systolic and/or diastolic heart failure. Additionally, acquired valvular diseases can contribute to the progression to end-stage heart failure.

Cardiomyopathies, including dilated, hypertrophic, and restrictive forms, may present at any age and may be idiopathic or genetic. Depending on the cardiomyopathy, HF may be primarily due to systolic or diastolic impairment or both, particularly at end-stage disease. In infants, cardiomyopathy is often secondary to metabolic or syndromic causes.

Myocarditis, most often viral in origin, is a leading acquired cause of pediatric HF [6]. Presentations range from mild symptoms to fulminant HF or cardiogenic shock [7,8,9]. The distinction between myocardial depression from sepsis and primary myocarditis is important, given potentially differing treatments and outcomes. Immune-mediated myocarditis following COVID-19 vaccination, although rare and usually self-limited, is also recognized. Persistent tachyarrhythmias and pacemaker-induced dyssynchrony are other reversible causes of myocardial dysfunction. Endocrine, nutritional, and rheumatologic causes must also be considered in the differential diagnosis.

## 3. Signs and Symptoms of Pediatric Heart Failure

HF in children is a clinical syndrome characterized by the heart’s inability to provide adequate cardiac output to meet the metabolic demands of the body. Unlike adults, pediatric patients rarely present with classic signs such as peripheral edema or orthopnea. Infants often manifest with nonspecific symptoms, including feeding intolerance, diaphoresis during feeding, and failure to thrive. Older children may present with fatigue, abdominal pain, and nausea—symptoms frequently misattributed to common pediatric illnesses. For example, recurrent or persistent vomiting in an infant may be misdiagnosed as gastroesophageal reflux; an older child with nausea, vomiting, and hepatomegaly may be referred for evaluation of suspected gastrointestinal pathology; and cough with tachypnea may be mistaken for a viral respiratory syndrome. These overlapping presentations can delay recognition and appropriate management of pediatric heart failure [10,11,12].

Physical examination in pediatric HF commonly reveals tachycardia, tachypnea, hepatomegaly, and a gallop rhythm. The presence of hypotension and peripheral edema typically signifies a decompensated state. Recognition of abnormal heart sounds, persistent tachycardia in an afebrile child, and, although less common, crackles on auscultation or extremity edema should prompt further evaluation, including chest radiography and echocardiography (Table 3).

Table 3 presents a recommended algorithm for the assessment and referral of patients with suspected heart failure. Measurement of circulating biomarkers, including brain natriuretic peptide (BNP) and N-terminal pro-brain natriuretic peptide (NT-proBNP), constitutes an important adjunctive test. BNP is a cardiac hormone released in response to myocardial stress and stretch, whereas NT-proBNP is an inactive fragment of the precursor molecule, favored in some centers due to its longer half-life. Both biomarkers are elevated in heart failure and are routinely utilized by emergency medicine, cardiology, and critical care providers for diagnosis and monitoring of therapeutic response. Additionally, BNP and NT-proBNP levels have prognostic value, with higher concentrations correlating with adverse outcomes [13]. Referral to emergency care is warranted for persistent feeding intolerance in infants, unexplained failure to thrive, and in older children, persistent abdominal symptoms with any signs of cardiovascular compromise. Educational resources such as the ACTION HF Handbook can aid both clinicians and families in recognizing early symptoms (https://myactioneducation.org, accessed on 8 June 2025).

## 4. Current Treatment Modalities for Children with Heart Failure

The treatment strategy for pediatric HF is multifaceted and highly dependent on the underlying etiology. Management may include pharmacologic therapy, surgical or catheter-based interventions, and in some cases, advanced therapies such as mechanical circulatory support or heart transplantation [3,7].

In cases of CHD, definitive treatment often involves surgical repair or palliation once the patient’s hemodynamic and nutritional status are optimized [3]. High-output states such as arteriovenous malformations may require embolization or surgical ligation. For valvular lesions, catheter-based interventions such as balloon valvuloplasty or surgical valve repair may be appropriate, depending on the severity and anatomical considerations.

Very few placebo-controlled randomized trials have been conducted in pediatric heart failure, resulting in limited direct evidence to guide pharmacologic therapy in this population. Consequently, most medications used by pediatric heart failure specialists are extrapolated from adult studies, with recommendations largely based on expert consensus [3,14]. In the United States, the only heart failure therapies approved by the Food and Drug Administration for pediatric use are sacubitril/valsartan and ivabradine—the latter indicated for heart rate control in children with persistent tachycardia despite beta-blocker therapy. In Europe, the European Medicines Agency has approved sacubitril/valsartan and enalapril for pediatric heart failure. Variability in drug availability and regulatory approvals across countries further complicates efforts to standardize pediatric heart failure treatment globally.

Pharmacologic therapy remains the cornerstone of management for children with cardiomyopathy or myocarditis and HF. Initial inpatient stabilization frequently includes diuretics to alleviate fluid overload, inotropes and inodilators to enhance myocardial contractility, and vasodilators to reduce afterload. Milrinone is widely used in the United States due to its combined inotropic and vasodilatory effects, including pulmonary vasodilation, which supports right ventricular function and reduces afterload to promote forward flow to the left ventricle. Levosimendan, an alternative inodilator more commonly employed internationally, similarly augments contractility and induces vasodilation across both pulmonary and systemic vascular beds. Additional agents such as epinephrine or dopamine may be used in acute decompensated states. Loop diuretics, primarily furosemide, are the most commonly administered agents for volume management, often supplemented by thiazide diuretics as needed. Close monitoring of electrolytes and renal function is essential during therapy. Long-term outpatient therapy commonly includes medications targeting the renin–angiotensin–aldosterone system (RAAS), such as ACE inhibitors (e.g., enalapril), angiotensin receptor blockers (e.g., valsartan), and mineralocorticoid receptor antagonists (e.g., spironolactone). Beta-blockers like carvedilol are often added for their long-term cardioprotective effects, despite limited pediatric-specific trial data [15]. A new class of medications known as angiotensin receptor–neprilysin inhibitor (sacubitril/valsartan) is also being utilized more recently [16].

Emerging therapies such as sodium-glucose co-transporter-2 (SGLT2) inhibitors are under investigation in pediatric populations after demonstrating substantial benefit in adult HF cohorts. Although digoxin use has declined, it may still offer symptomatic benefit in select patients with persistent symptoms [17]. Digoxin has a narrow therapeutic index and should be administered at low doses targeting serum concentrations between 0.5 and 1.0 ng/mL (0.6–1.3 nmol/L) to minimize the risk of toxicity. Adverse effects include arrhythmias, increased mortality, gastrointestinal symptoms, and visual disturbances. Additionally, hypokalemia potentiates digoxin toxicity, necessitating careful monitoring of electrolyte levels during therapy.

Close monitoring of renal function and electrolytes is imperative, particularly when combining renin–angiotensin–aldosterone system (RAAS) inhibitors with diuretics. Importantly, captopril is preferred over enalapril in neonates and young infants due to a more favorable safety profile in this age group. Table 4 summarizes commonly used medications in pediatric HF management.

This individualized, etiology-driven approach enables a tailored treatment plan that balances efficacy, safety, and quality of life for each pediatric HF patient.

## 5. Advanced Heart Failure Therapies

When conventional medical therapies are insufficient to manage symptoms or halt disease progression, advanced therapies may be indicated. These include mechanical circulatory support with devices such as extracorporeal membrane oxygenation (ECMO) or ventricular assist devices (VADs), and ultimately, heart transplantation.

Extracorporeal membrane oxygenation (ECMO) is typically employed in cases of acute decompensation or cardiogenic shock requiring rapid circulatory support. It is also used postoperatively in unstable patients as a bridge to recovery of cardiac and respiratory function. ECMO’s advantage lies in its rapid deployment and capacity to provide simultaneous cardiac and respiratory support, although its use is generally limited to the short term. For patients with a more gradual progression of heart failure, ventricular assist devices (VADs) are preferred due to their suitability for longer-term support and enhanced hemodynamic stabilization. For smaller children or infants, VADs are generally extracorporeal due to size limitations, necessitating prolonged hospitalization. Older and larger children may be candidates for implantable VADs, potentially allowing hospital discharge and reintegration into school or other activities. A multidisciplinary team—including cardiology, cardiothoracic surgery, intensive care, and palliative care—is essential in assessing candidacy for VAD support. Risks include thromboembolism, bleeding, infection, and neurologic events, which can impact the feasibility of eventual transplantation [24].

Heart transplantation remains the definitive therapy for select patients with end-stage heart failure; however, it is a palliative intervention rather than a cure, as it replaces one set of medical challenges with another. Lifelong immunosuppression is required, and overall life expectancy remains reduced compared to the general population. Although survival following pediatric heart transplantation has improved substantially, outcomes are influenced by multiple patient-specific factors. Complications such as primary graft failure, rejection, and cardiac allograft vasculopathy continue to limit long-term survival, with some patients ultimately requiring re-transplantation [25]. Success hinges on careful patient selection, strong social support, and stringent adherence to post-transplant regimens. Psychosocial evaluation and family engagement are essential components of the transplant process. For families who choose not to pursue VAD or transplant—or for whom these options are not feasible—care should focus on quality of life. Oral and parenteral inotropic therapies, such as long-term milrinone infusions, may be considered to reduce symptoms and support home-based care. Early integration of palliative care and, when appropriate, hospice services can provide symptom relief and emotional support, aligning treatment with family goals [26].

## 6. Key Supportive Considerations in Pediatric Heart Failure

### 6.1. Immunization and Infection Risk Management

Children with HF, particularly those awaiting transplant or on immunosuppressive therapy, are at increased risk for infection. Vaccinations are essential to reduce morbidity and mortality, especially against encapsulated organisms, influenza, and respiratory viruses such as RSV and COVID-19 [27,28,29,30]. Families should be educated about the timing of immunizations in relation to transplant listing, particularly with live vaccines. Consultation with transplant infectious disease teams is advised when planning immunizations in actively listed patients [26,27].

### 6.2. Iron Deficiency and Nutritional Support

Iron deficiency is a common comorbidity in pediatric HF and is independently associated with worse outcomes, including increased need for VAD placement and transplantation [31]. Screening should include measurement of serum ferritin, transferrin saturation, and iron levels, as anemia may be absent in some cases despite iron deficiency. Conversely, a “normal” hemoglobin level in a chronically cyanotic child is likely indicative of anemia. Oral iron therapy is often inadequate; intravenous iron may be required, particularly in symptomatic children or those with advanced disease [32,33].

### 6.3. School and Physical Activity

Children with HF benefit from structured care plans that support consistent school attendance and engagement in age-appropriate physical activity [34]. Collaboration between healthcare providers and schools is essential to implement accommodations through Section 504 plans or individualized education programs (IEPs). Depending on symptom severity, accommodations may include extended time to walk between classes, elevator use in place of stairs, reduced classroom hours, or fewer days of attendance. Thorough assessment of available school resources and supports is critical when developing and implementing these plans. Decisions regarding participation in recess, physical education, and competitive sports should be made in consultation with the child’s cardiologist to balance the benefits of activity with the risk of deconditioning and adverse events. Activity restrictions are individualized based on exercise testing, echocardiographic findings, and arrhythmia risk, as well as the severity of symptoms and lesion-specific considerations [35]. Most children with mild left ventricular systolic dysfunction who are minimally symptomatic and free of significant arrhythmias may be allowed full participation through shared decision-making with the family. As with all school settings, the availability of automated external defibrillators (AEDs) and personnel trained in cardiopulmonary resuscitation (CPR) is essential. Minimizing physical deconditioning is vital for both physical and psychosocial well-being.

### 6.4. Mental Health and Family Support

Pediatric HF has profound effects on the emotional well-being of patients and families. Children may experience depression, anxiety, or adjustment disorders, particularly if dependent on VADs or awaiting transplant [36,37]. Caregivers and siblings may also exhibit symptoms of traumatic stress or emotional burnout [38,39]. Mental health screening of children and their parents should be part of routine pediatric care, with referrals to psychology and social work services as needed. Empowering caregivers through education, self-care guidance, and psychosocial support, including local support groups which vary by country, city, and community, improves family resilience and outcomes.

### 6.5. Pediatric Primary Care Monitoring

The primary pediatric care provider plays a pivotal role in the longitudinal follow-up of children with heart failure, as outlined in the preceding sections. At each encounter, weight trends should be carefully assessed: excessive weight gain may signify fluid retention, whereas poor weight gain may reflect inadequate caloric intake or progression of heart failure despite apparently sufficient intake. Any concerning deviation from baseline should prompt immediate communication with the heart failure team. Minor infections are generally managed according to standard pediatric protocols; however, in patients actively listed for heart transplantation, the transplant team should be notified promptly, as donor organ offers are typically not accepted during episodes of acute illness.

## 7. Conclusions

Optimizing outcomes in pediatric HF begins with early recognition of symptoms, accurate diagnosis, and timely initiation of evidence-based therapy. Primary care providers are uniquely positioned to serve as the bridge between families and subspecialty care teams. Their role in preventive care, chronic disease management, and education cannot be overstated. By understanding the complexities of pediatric HF and working collaboratively with cardiology teams, primary care providers can help ensure that children receive timely, appropriate, and family-centered care throughout the disease continuum.

## Figures and Tables

**Table 1:** Causes of pediatric heart failure.

Myocardial Abnormality	Structural Heart Disease
**Primary** Arrhythmogenic cardiomyopathy Dilated cardiomyopathy Hypertrophic cardiomyopathy Non-compaction cardiomyopathy Restrictive cardiomyopathy Chemotherapy-related Dystrophinopathy-related Mitochondrial cardiomyopathy	**Congenital Heart Disease** Unrepaired coarctation of the aorta or chronic aortic stenosis Unrepaired shunting lesions (left to right shunt lesions such as atrial or ventricular septal defect, patent ductus arteriosus) causing over-circulation End-stage biventricular congenital heart disease End-stage single ventricle congenital heart disease Arteriovenous Malformations
**Secondary** Arrhythmia-induced cardiomyopathy Myocarditis Chemotherapy (immune checkpoint inhibitor-related) Post-vaccine Rheumatologic Sepsis-related cardiomyopathy Viral Metabolic Glycogen Storage disorder Lysosomal storage disorder Endocrinologic Adrenal insufficiency Hypoparathyroidism Hypothyroidisim Nutritional Iron deficiency Selenium deficiency Thiamine deficiency	**Acquired Heart Disease** Acute rheumatic fever Kawasaki disease Rheumatic heart disease Endocarditis

**Table 2:** Causes of pediatric heart failure by age.

Age Group	Likely Causes of Heart Failure
**Infants (Birth to 12 months)**	-Congenital heart disease: critical left-sided obstructive lesions, shunt lesions (e.g., VSD, AVSD, PDA), arteriovenous malformations -Non-structural causes: birth asphyxia, sepsis, myocarditis, metabolic disorders, primary cardiomyopathy, incessant tachycardia, infant of diabetic mother
**Toddlers (1 to 3 years)**	-Congenital heart disease: residual or repaired lesions, valve disease -Non-structural causes: myocarditis, Kawasaki disease, metabolic disorders, primary cardiomyopathy, tachycardia-induced cardiomyopathy
**Older Children (>3 years)**	-Congenital heart disease: palliated single ventricle physiology, chronic valve disease, pacing-induced cardiomyopathy -Non-structural causes: primary cardiomyopathy, inflammatory diseases (myocarditis, Kawasaki disease, rheumatic fever), autoimmune disorders, chemotherapy-related cardiotoxicity

**Table 3:** Diagnostic approach for pediatric heart failure.

Category	Details
**History**	**Infants:** feeding difficulty, early tiring with feeding, tachypnea ± feeding, diaphoresis/clamminess ± feeding, frequent irritability, pale/ashy/bluish discoloration, failing to thrive **Children/Adolescents:** shortness of breath, decreased appetite, persistent nausea/vomiting, fatigue, decreased activity tolerance
**Family History**	Heart failure, enlarged/thick hearts, cardiac medications, pacemakers, defibrillators, cardiomyopathy, sudden death, unexplained fainting, arrhythmias
**Physical Exam**	Length/height, weight (and trend), vital signs, tachypnea, retractions, rales, increased/displaced cardiac impulse, abnormal heart sounds (loud or single S2, murmur, click, rub, gallop), skin color/temperature, liver size, pulse characteristics
**Further Evaluation**	**If mild symptoms, no distress signs:** -CXR (check cardiomegaly, pulmonary vascularity) -ECG (check for abnormalities) **If persistent symptoms/signs with normal tests:** -Refer to cardiology -Echocardiogram (structure and function) -Advanced/invasive testing as needed case by case
	**If symptoms with any signs of distress:** -Send to emergency room for stabilization and evaluation -CXR, ECG, Echocardiogram -Additional testing case by case

**Table 4:** Doses and side effects of commonly used pediatric heart failure drugs [18,19,20,21,22,23].

Medication	Initial Dose	Target	Max Dose	Side Effects
Captopril (ACEI)	0.33 mg/kg/dose every 8 h	1 mg/kg/dose every 8 h	2 mg/kg/dose every 8 h (50 mg/dose)	Angioedema, Cough, Hypotension, Hyperkalemia, Renal Dysfunction
Enalapril (ACEI)	0.05–0.1 mg/kg/dose every 12 h	0.2 mg/kg/dose every 12 h	0.5 mg/kg/dose every 12 h (20 mg/dose)	Angioedema, Cough, Hypotension, Hyperkalemia, Renal Dysfunction
Lisinopril (ACEI)	0.1 mg/kg/dose every 24 h	0.4 mg/kg/dose every 24 h	0.8 mg/kg/dose every 24 h (40 mg/dose)	Angioedema, Cough, Hypotension, Hyperkalemia, Renal Dysfunction
Losartan (ARB)	0.5 mg/kg/dose every 24 h	1 mg/kg/dose every 24 h	1.4 mg/kg/dose every 24 h (150 mg/dose)	Hypotension, Hyperkalemia, Hypoglycemia, Renal Dysfunction
Sacubitril-Valsartan (ARNI) (Low or no prior ACEI/ARB)	0.8 mg/kg/dose every 12 h	3.1 mg/kg/dose every 12 h	3.1 mg/kg/dose every 12 h (97–103 mg/dose)	Angioedema, Hyperkalemia, Hypotension, Renal Dysfunction
Sacubitril-Valsartan (ARNI) (Prior ACEI/ARB)	1.6 mg/kg/dose every 12 h	3.1 mg/kg/dose every 12 h	3.1 mg/kg/dose every 12 h (97–103 mg/dose)	Angioedema, Hyperkalemia, Hypotension, Renal Dysfunction
Carvedilol (Beta-Blocker)	0.05 mg/kg/dose every 12 h	0.5 mg/kg/dose every 12 h	1 mg/kg/dose every 12 h (50 mg/dose)	Hypotension, Bradycardia, Hypoglycemia
Metoprolol Tartrate (IR)(Beta-Blocker)	0.25 mg/kg/dose every 12 h	0.5 mg/kg/dose every 12 h	1 mg/kg/dose BID (100 mg/dose)	Hypotension, Bradycardia, Hypoglycemia
Metoprolol Succinate (XL)(Beta-Blocker) *	12.5–25 mg/dose every 24 h	100 mg/dose every 24 h	200 mg/dose every 24 h	Hypotension, Bradycardia, Hypoglycemia
Spironolactone (MRA)	1–3 mg/kg/day divided every 8–12 h	3 mg/kg/day	6 mg/kg day (100 mg/day)	Hyperkalemia, Gynecomastia
Eplerenone (MRA) *	25 mg/dose every 24 h	25 mg/dose every 24 h	50 mg/dose every 24 h	Hyperkalemia
Dapagliflozin (SGLT2-i) +	0.1 mg/kg/dose every 24 h	0.1–0.2 mg/kg/dose every 24 h	0.2 mg/kg/dose every 24 h (10 mg/dose)	Hypotension, UTI, Hypoglycemia
Empagliflozin (SGLT2-i) *	5 mg/dose every 24 h	5 mg/dose every 24 h	10 mg/dose every 24 h	Hypotension, UTI, Hypoglycemia

(Adapted with permission from the ACTION learning network [18]. 2023, ACTION Learning Network). * Only available as a tablet, so should only be prescribed for patients able to swallow whole dosage forms. + Requires family instruction on how to crush the tablet and administer to the patient.

## Abbreviations

The following abbreviations are used in this manuscript:

HF	Heart failure
CHD	Congenital heart disease
VAD	Ventricular assist device
PCP	Primary care provider
ACTION	Advanced Cardiac Therapies Improving Outcomes Network
FON	Fontan Outcomes Network
MRA	Mineralocorticoid receptor antagonist
ARNI	Angiotensin receptor–neprilysin inhibitor
SGLT2	Sodium-glucose co-transporter 2
